# Successful use of Fenfluramine in super-refractory status epilepticus in a patient with tuberous sclerosis complex and Lennox-Gastaut syndrome

**DOI:** 10.1016/j.ebr.2024.100697

**Published:** 2024-07-21

**Authors:** Thorsten Wildermann, Felicitas Becker, Sarah Jesse, Hartmut Baier, Jan Wagner

**Affiliations:** a Department of Neurology, University of Ulm, Oberer Eselsberg 45, 89081 Ulm, Germany; b ZfP Südwürttemberg Department Epileptology, Weingartshofer Str. 2, 88214 Ravensburg, Germany

**Keywords:** Super-refractory status epilepticus, Tuberous sclerosis complex, Lennox-Gastaut syndrome, Fenfluramine

## Abstract

A 24-year-old female patient with pre-existing refractory epilepsy caused by tuberous sclerosis (TSC) and electroclinical features of Lennox-Gastaut syndrome (LGS) was referred to our hospital from an external clinic. Upon arrival, she presented with super-refractory status epilepticus (SRSE) since anaesthetics had already been used in the referring clinic. Despite various changes in ASM-treatment and continuous administration of anaesthetics for more than two weeks, SRSE could not be terminated. On treatment day 24, we started Fenfluramin (FFA) which was soon titrated to a dose of 0,7 mg/kg/day. A few days after beginning the treatment with FFA, EEG and clinical situation improved dramatically. The following 6 weeks of treatment went without reported seizures. This case illustrates the successful use of FFA in SRSE in TSC and LGS and, to the best of our knowledge, represents the first report of FFA in this clinical context.

## Highlights

• Successful use of Fenfluramine in super-refractory status epilepticus.
• Patient with tuberous sclerosis complex.
• Electroclinical features of Lennox-Gastaut syndrome.
• No serious adverse events, no pulmonary or cardiac problems during follow-up.

## 1 Introduction

Super-refractory status epilepticus (SRSE) is defined as status epilepticus (SE) that remains uncontrolled within 24 hours despite third-line anti-seizure therapy or reoccurs/persists after cessation of third-line therapy [1]. Due to the small number of patients developing SRSE, current therapy concepts are mostly defined by retrospective data, case reports or clinical experience. The severity of this disease is reflected by an in-hospital mortality of around 24 % and bad functional outcomes of patients at discharge (only 25 % with modified Ranking Scale (mRS) < 2) [2]. The predominant etiologies seem to be acute cerebral events, whereas known epilepsies account for less than 20 % of SRSE patients [3]. Lennox-Gastaut syndrome (LGS) is considered a developmental and epileptic encephalopathy (DEE). Main features are drug-resistant seizures (i.e. tonic/atonic seizures), intellectual disability and spike-slow-wave/generalised paroxysmal fast activity in EEG. Since LGS is a description of electroclinical features, it can have different underlying etiologies. Drug resistance and status epilepticus is common in LGS [4]. Tuberous sclerosis complex (TSC) is a genetic disorder affecting different organ systems. Genetic mutations in TSC1/2 gene cause a dysregulation in the mTOR (mammalian Target of Rapamycin) – pathway. Epilepsy is a typical neurological symptom in TSC often starting in very young children (<1 year of age). Epileptic spasms are a common feature associated with TSC and are seen in up to 50 % of TSC patients [5]. Though LGS is a common syndrome in TSC patients >1 year of age data on the coincidence of TSC and LGS is scarce. In a group of 21 TSC patients followed in a prospective study in Egypt, 35 % developed LGS during a 2–4 year follow-up period [6], [7]. Fenfluramine (FFA) was initially approved for the add-on treatment of seizures associated with Dravet syndrome (DS) and recently for LGS . There is evidence from animal models that mechanisms of seizure reduction by FFA may be connected to certain subtypes of 5HT-receptors and σ1-receptors though further evaluation is needed [8], [9]. To date, four randomized, placebo controlled studies have evaluated the safety and efficacy of FFA in 615 patients. Three studies were performed on a cumulative number of 352 DS patients, while one study was performed on 263 LGS patients. FFA demonstrated its efficacy with a reduced number of seizures in comparison to placebo (up to 50–60 % greater reduction than placebo) [10], [11], [12], [13]. Regarding the use of FFA in SE/SRSE, only case reports exits in DS [1], [14]. To our knowledge, this is the first case report on the use of FFA in SRSE in LGS and TSC.

## 2 Case presentation

**Fig. 1:**
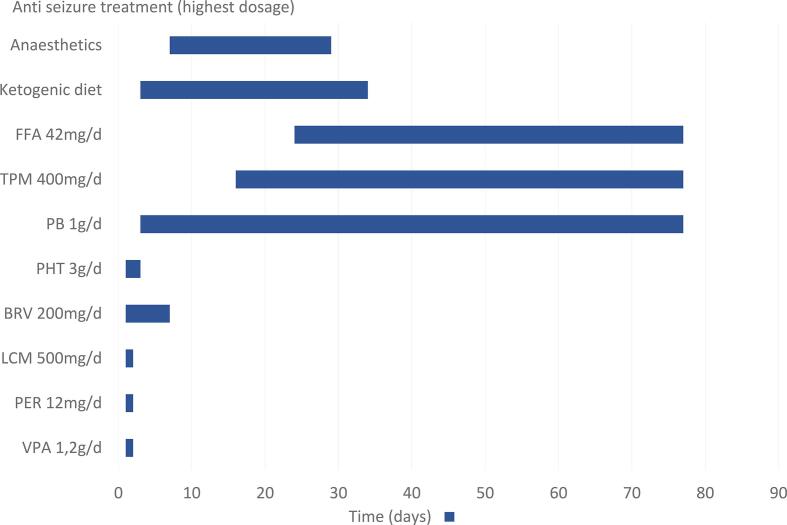
Showing the-anti seizure treatment given during the stay in our hospital including ASM and ketogenic diet. Illustrating highest dosage and application period in milligramms/day (mg/d) and grams/day (g/d). Abbreviations: FFA – fenfluramine, TPM – topiramate, PB – phenobarbital, PHT – phenytoin, BRV – brivaracetam, LCM – lacosamide, PER – perampanel, VPA- valproic acid. Anaesthestics used included: esketamin, propofol, diazepam, isoflurane.

**Fig. 2:**
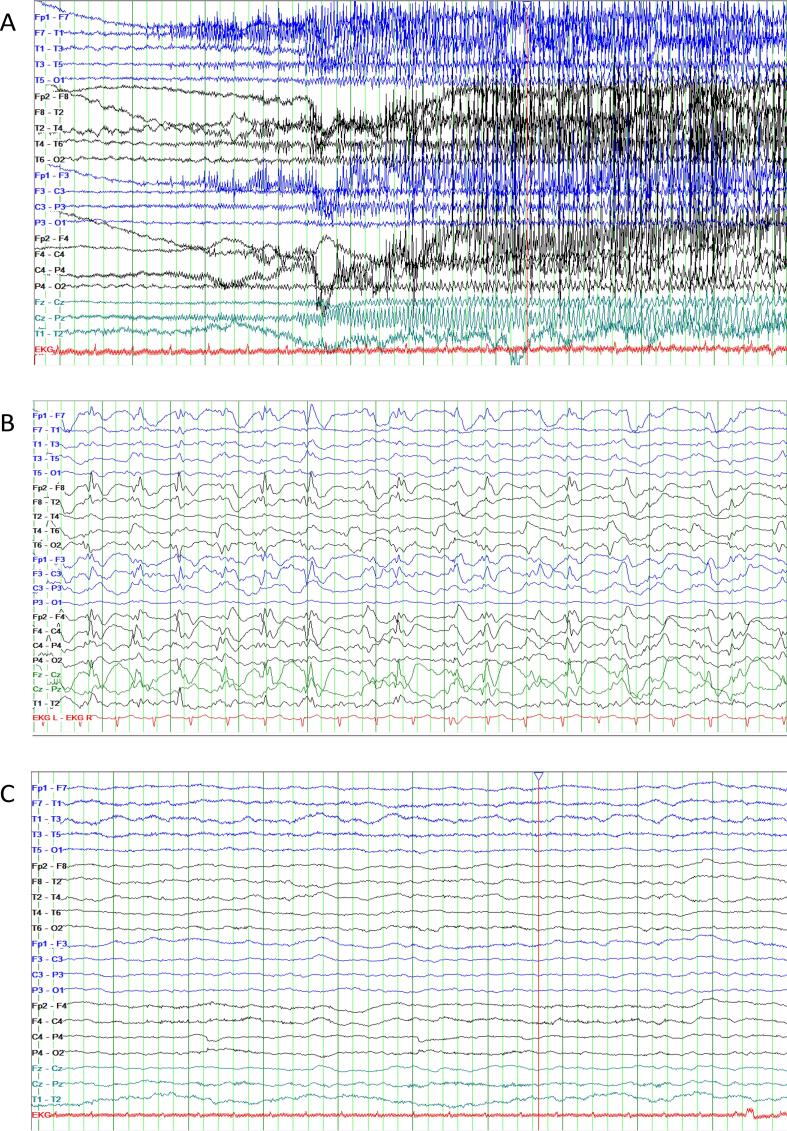
Panel A. Initial EEG upon arrival showing fast ictal activity in the central region and muscle artefacts during a tonic seizure. Panel B. Treatment day 7 – EEG showing periodic sharp-slow-wave/periodic discharges mainly in the frontocentral regions. Panel C. EEG after initiation of FFA showing diffuse slowing. No epileptiform discharges. All EEGs displayed at 7 µV/mm, HFF 70 Hz, LFF 0.5 Hz, 30 mm/sec (millimeters/second).

**Fig. 3:**
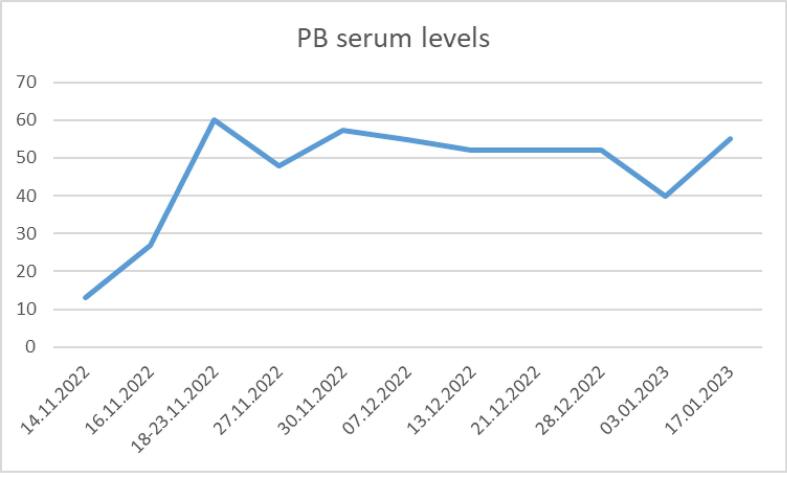
Showing serum levels of PB during the hospital stay in mg/l. Abbreviations: PB – Phenobarbital.

A 24-year-old female patient was transferred to our clinic on 10.11.2022 with SRSE. The patient had a history of epileptic spasms starting at the age of 3 months. She was consequently treated with vigabatrin for 6 months. At the age of one the diagnosis of tuberous sclerosis complex was made. Genetic testing in 2017 confirmed the clinical diagnosis revealing a mutation in the TSC2 gene. Over time the patient developed drug-resistant epilepsy with tonic, myoclonic and generalised tonic-clonic seizures (GTCS). Longer periods of seizure freedom were never achieved despite usage of multiple combinations of anti-seizure medications (ASMs) (see Table 1). Initially, she was admitted to an external clinic with recurring GTCS under treatment with lacosamide (LCM) 500 mg/day and brivaracetam (BRV) 200 mg/day. According to the parents, her usual seizure frequency at that time was around 2–3 seizures/d (tonic or GTCS). After initial unsuccessful treatment with midazolam, valproic acid (VPA) and perampanel (PER) was quickly added. From 03.11.2022–09.11.2022 anaesthetics were applied (propofol), resulting in a cessation of the seizures. As propofol was reduced, seizures reoccurred rapidly, prompting the transfer to our epilepsy center. Upon arrival, the patient was awake and presented with continuous clonic movements in the upper and lower extremities. Additionally, she experienced three tonic-clonic seizures within the first 15 min without regaining consciousness, confirming the diagnosis of SRSE. A complementary EEG study showed recurring seizure patterns. She was under ASM treatment with VPA (1.2 g/day, serum level >90 mg/l), LCM (500 mg/day) BRV (200 mg/day), and PER (12 mg/day). Aside from VPA, ASM levels were not measured in the external clinic. Initial laboratory testing included blood count, potassium, sodium, chloride, INR, PTT, creatinine, serum glucose, c-reactive-protein and blood gas analysis. The aforementioned tests were performed on a regular basis and did not reveal a reason for SRSE. In addition, regular serum levels of ASM medication were measured (details see supplement Table 1). An MRI was performed showing typical changes in TSC with cortical and subcortical tubera as well as SEGA (subependymal giant cell astrocytoma) (supplement 2). In comparison to the previous MRI, no relevant changes could be detected. In conclusion, no certain trigger for SRSE could be identified. A therapy with intravenous phenytoin (PHT) was established (loading dose of 1.5 g over 30 min followed by 750 mg over 12 h), while VPA and LCM were discontinued. Despite sufficient PHT serum levels (day 1: 13.3 mg/l, d2: 21.5 mg/l, d3 27.8  g/l, d10 < 5 mg/l), SE persisted. Consequently, we transitioned to PHT to phenobarbital (PB) and added ketogenic diet (KD). An overview of the ASM therapies used as well as ASM levels of PB during the stay in our hospital is provided in Fig. 1 and Fig. 3. After one week of therapy, SE could not be terminated so we decided to try anaesthetics starting with diazepam on day 7. From day 6 to day 18 continuous EEG-monitoring was recorded, after day 18 regular routine EEG studies were done. EEG changes included generalised fast activity and bilateral spike-slow-wave activity. Despite additional treatment with different anaesthetics, sufficient seizure suppression or burst-suppression >24 h couldn’t be achieved until day 24, despite high serum levels of PB (from day 7 after initiation onwards serum levels never dipped below 40 mg/l, with most measurements >50 mg/l), sufficient ketosis (serum ketone levels >5 mg/dl) and the start of topiramate (TPM, 400 mg/day, serum levels measured once on 14.12.2022 with low levels of 1.8 mg/l). Therefore we decided to add an off-label medication with FFA on day 24. Beforehand an echocardiogram was performed ruling out changes in heart valves or pulmonary hypertension. Approval of the parents was given prior to the administration of FFA. FFA dosage was increased to a peak dose of 42 mg/day within 8 days, which equalled around 0,7mg/kg. FFA was administered via a nasogastral tube. During the first five days of treatment, the patient still presented with multiple seizures per day. After day 6 of FFA treatment, no more seizures were observed. SRSE ceased, the EEG improved, and she regained consciousness. The EEG changes can be seen in Fig. 2. Even oral feeding could slowly be established but stayed insufficient during the further hospital stay so that a percutaneous endoscopic gastrostomy (PEG) was placed on 13.01.2023. The patient was referred to a neurologic hospital near her hometown on 25.01.2023, having been free from any seizures during the last weeks in our clinic with the following ASM combination: FFA, TPM, PB. During the treatment, recurring fever episodes were detected which often led to an antibiotic treatment. For some of the episodes, no infectious cause could be identified, so a connection with FFA was suspected, since it is a known side effect. Following the stay in our hospital, she stayed for 3 months in the epilepsy centre Weissenau. Here, clinically discrete seizures with eyelid-myoclonus and rare tonic seizures reoccurred. GTCS or SE did not reoccur. FFA was reduced to 21 mg/day. However, the recurrent fever episodes persisted. Everolimus (EVR) was reintroduced as ASM and disease-modifying drug. Unfortunately, after the long hospital stay the patient stayed bedridden. The patient was last seen in August 2023 for an echocardiogram, which still showed no signs of valve changes or pulmonary hypertension. The last available medication regimen included FFA 21 mg/day, PB 200 mg/day, TPM 900 mg/day, LCM 400 mg/day and EVR 5 mg/day. Follow-up visits with the patient occurred in the epilepsy centre Weissenau. Last information acquired in March 2024 reavealed overall improvement in seizure frequency to around 1–2 tonic seizures per month, altough exacerbations of seizures happened in November 2023 and February 2024 resulting in additional doses of PB and LCM. Hospitalisation did not occur after discharge from our clinic.

**Table 1:** History of anti-seizure medication including highest daily dosages given in milligrams/day.

Anti-seizure medication	Highest dosage in milligrams/day	Time/duration
Vigabatrine	900 mg/d	1998
Sulthiame	n.a.	2000
Methosuximide	375 mg/d	Before 2003
Pregabalin	n.a.	Before 2003
Primidone	n.a.	Before 2003
Zonisamide	n.a.	Before 2003
Topiramate	900 mg/d	2000, 2022-today
Phenytoine	1500 mg/d	2008–2020
Levetiracetam	3000 mg/d	2010–2020
Valproic acid	1500 mg/d	2010
Lacosamide	500 mg/d	2010, 2016, 2020, 2023
Clobazam	20 mg/d	2014
Rufinamide	3200 mg/d	2015
Cannabidiol	1580 mg/d	2020
Everolimus	2,5mg/d	2020,2023
Brivaracetam	100 mg/d	2020–2022
Perampanel	12 mg/d	2022–2023
Phenobarbital	600 mg/d	2022-today

## 3 Discussion

To our knowledge, this is the first case report for the use of FFA in SRSE in TSC or LGS. Few other case reports showed an effect of FFA on SE/RSE in patients with DS and LGS [1], [14], [15], [16]. Two studies demonstrated a lasting effect of reduced SE and/or seizure frequency up to two years after discharge, meanwhile the other reports had short follow-up times (<2 months) [1], [14]. After the unsuccessful usage of multiple ASMs as well as anaesthetics and ketogenic diet that happened in accordance with the German guidelines for treatment of status epilepticus, we discussed the remaining treatment options for our patient. To do so, we reviewed the clinical and electroencephalographic features, as well as the medical history (as far as attainable), leading to the conclusion that, in consequence to the known TSC, the patient fulfilled the criteria for LGS. As mentioned in the introduction, FFA has proven to reduce seizures in LGS patients, although knowledge on the use in SE/SRSE remains scarce [10]. Therefore, we decided to try FFA after discussion with the family. We debated starting zonisamide, but due to its similarities to TPM, we ultimately decided against it. We also ruled out immunomodulatory therapy as an alternative treatment because no signs of encephalitis existed, and the patient did not have new onset refractory status epilepticus (NORSE). Upon introduction of FFA, clinical and electroencephalographic improvement was observed on treatment day 6, making a correlation highly probable. This also fits well with the elimination half-life of FFA, which is around 20 hours, leading to a serum steady-state in most patients after 4–5 days [17]. This is in accordance with the aforementioned case reports, where cessation was seen within a week after the initiation of FFA [14]. Eight days prior to the start of FFA, the last change in ASM-regiment was the addition of TPM, making a singular effect of TPM improbable. The low serum levels of TPM also support this hypothesis. High serum concentrations of PB measured at the time FFA was introduced might have played a role in termination of SRSE, though serum levels were >50 mg/l for more than 2 weeks prior to the start of FFA without adequate effects on SRSE. A combined effect of FFA, TPM and PB is possible but can neither be proven nor discredited. Since we rapidly increased the dosage of FFA in our case, no statement can be made on the efficacy of lower doses. Regarding the patients follow-up with exacerbations of seizures but overall reduced seizure frequency it could be speculated that the reduction of FFA to the approved dosage was associated with these relapses.

Overall, our case suggests the possibility of a successful use of FFA in SE/SRSE in TSC/LGS and should be considered in these patients. Still, further data and time is needed to confirm or discredit the usefulness in regard to SE/SRSE in the future.

## Ethical statement

The treatment was in accordance with hospital policy and the patient’s family/legal guardian was informed and consented to the treatment with fenfluramine.

## Declaration of competing interest

The authors declare the following financial interests/personal relationships which may be considered as potential competing interests: PD Dr. J. Wagner received travel reimbursements and speakers fees from UCB. Felicitas Becker received travel reimbursements from UCB. Dr. H. Baier received speaker fees from UCB, Jazz Pharmaceuticals and Angelini Pharma.
