# Supplementary material for: Successful use of Fenfluramine in super-refractory status epilepticus in a patient with tuberous sclerosis complex and Lennox-Gastaut syndrome
Source: Epilepsy Behav Rep. 2024 Jul 21;27:100697. doi: 10.1016/j.ebr.2024.100697 (PMC11326921; doi:10.1016/j.ebr.2024.100697)
Supplement: Supplementary Data 2 [file mmc2.docx]

Supplement 2:

MRI from 22.11.2022 with exemplary pictures showing typical TSC associated changes


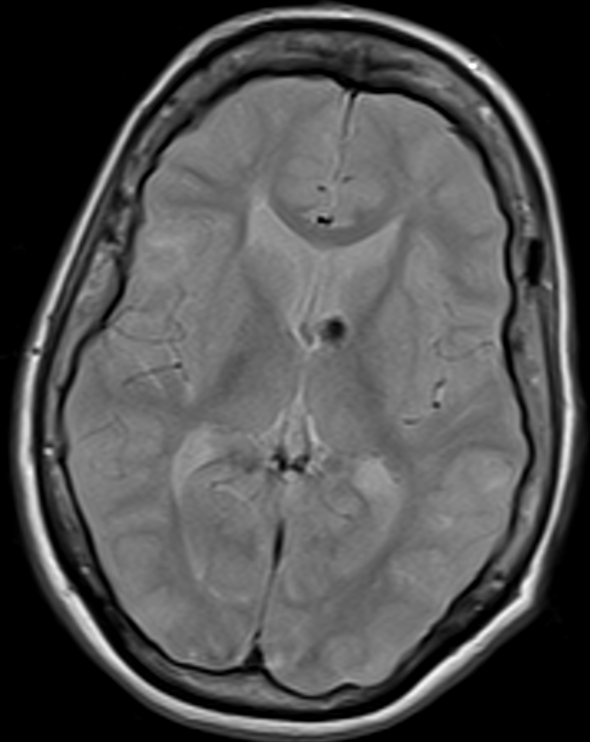


T2*-weighted image showing a periventricular SEGA (subependymal giant cell astrocytoma)


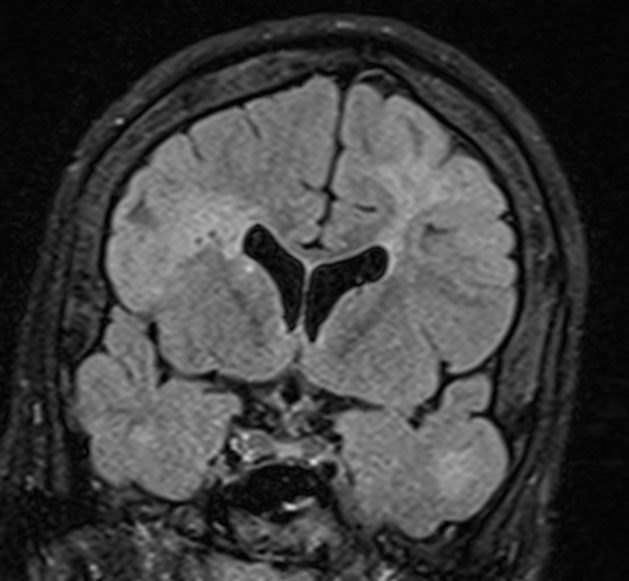


FLAIR image showing cortical und subcortical tubera
